# Ontario Veterinary College

**Published:** 1892-02

**Authors:** 


### ONTARIO VETERINARY COLLEGE.

### DECEMBER EXAMINATIONS.

The Board of Examiners met at the College on Tuesday, December 23. There were present the following veterinary surgeons: Messrs. Wilson, of London; Elliott, of St. Catherines; Cowan, of Galt; O'Neil, of London; W. Shaw, of Dayton, Ohio, and Loyd, of Newmarket. Many visitors were present and showed great interest in the examinations, among whom may be mentioned Professor Law, of Cornell University, Ithaca, N. Y.; D. McArthur, V. S., of Ailsa Craig, Ont.; J. T. Duncan, M.D., V.S., of the Ontario Veterinary College; S. Sisson, V. S., of the College; J. Thorburn, M. D., also of the College; D. Hamilton, V. S., of Harriston; W. Kidd, V.S., of Mitchell. The examinations were carried out in a very searching manner, the names of the successful students being given below.

On the following day, the 24th of December, was held the annual meeting of the Ontario Veterinary Medical Association, when a large number of veterinary surgeons were in attendance. The retiring President, W. Gibb, Esq., V. S., of St. Mary's, carried on the business with courtesy and ability. Among other matters discussed was a paper on Hog Cholera by W. Cowan, V. S., of Galt. Professor Law, of Cornell University, gave his views on the matter. Another interesting paper was by Mr. Wende, V. S., of Buffalo, on Poisoning by the Castor Oil Bean.

After professional topics had been well discussed, it was determined to hold a meeting in the City of London (Ontario) during the month of June, to which all members are invited. At the election of officers, Duncan McArthur, Esq., V. S., of Ailsa Craig, Ont., was unanimously elected President for 1892.

Graduating Students—Edgar A. Bogart, Kettleby, Ont.; Thomas H. Brooke, Don, Ont.; George C. Brownbridge, Brampton, Ont.; James Conrad Carter, Gowanda, N. Y.; James H. Chesney, Brucefield, Ont.; John M. Colvin, Wingham, Ont.; Thomas A. Corsant, Ilderton, Ont.; Adam Elgas, Hartford, Mich.; William Elliott, Delhi, N. Y.; Joseph Fotheringham, Mason City, Iowa; George H. Gibb, St. Mary's, Ont.; Richard T. Kidd, Listowel, Ont.; John Edwin Law, Ithaca, N. Y.; Charles F. Leslie, York, Neb.; B. B. Myers, Linville, Va.; Bernard R. Poole, England; Robert E. Stevenson, Toronto, Ont.; Jacob Wilhelm, Shakespeare, Ont.; Louis A. Willson, Eglington, Ont.; George E. Young, Toledo, Ohio.

The following have passed primary examinations only in the subjects stated: Asa A. Brown, Alfred T. Goldie, in anatomy; Horace Panet, James A. Wake, in materia medica.

